# Complementary Use of Carbohydrate Antigens Lewis a, Lewis b, and Sialyl-Lewis a (CA19.9 Epitope) in Gastrointestinal Cancers: Biological Rationale towards a Personalized Clinical Application

**DOI:** 10.3390/cancers12061509

**Published:** 2020-06-09

**Authors:** Rossella Indellicato, Aida Zulueta, Anna Caretti, Marco Trinchera

**Affiliations:** 1 Department of Health Sciences, University of Milan, 20142 Milano, Italy; rossella.indellicato@unimi.it (R.I.); aida.zulueta@unimi.it (A.Z.); anna.caretti@unimi.it (A.C.); 2 Department of Medicine and Surgery, University of Insubria, 21100 Varese, Italy

**Keywords:** tumor marker, glycosyltransferase, pancreatic ductal adenocarcinoma, colorectal cancer, selectin ligand

## Abstract

Carbohydrate antigen 19.9 (CA19.9) is used as a tumor marker for clinical and research purposes assuming that it is abundantly produced by gastrointestinal cancer cells due to a cancer-associated aberrant glycosylation favoring its synthesis. Recent data has instead suggested a different picture, where immunodetection on tissue sections matches biochemical and molecular data. In addition to CA19.9, structurally related carbohydrate antigens Lewis a and Lewis b are, in fact, undetectable in colon cancer, due to the down-regulation of a galactosyltransferase necessary for their synthesis. In the pancreas, no differential expression of CA19.9 or cognate glycosyltransferases occurs in cancer. Ductal cells only express such Lewis antigens in a pattern affected by the relative levels of each glycosyltransferase, which are genetically and epigenetically determined. The elevation of circulating antigens seems to depend on the obstruction of neoplastic ducts and loss of polarity occurring in malignant ductal cells. Circulating Lewis a and Lewis b are indeed promising candidates for monitoring pancreatic cancer patients that are negative for CA19.9, but not for improving the low diagnostic performance of such an antigen. Insufficient biological data are available for gastric and bile duct cancer. Studying each patient in a personalized manner determining all Lewis antigens in the surgical specimens and in the blood, together with the status of the tissue-specific glycosylation machinery, promises fruitful advances in translational research and clinical practice.

## 1. Introduction

The outcome of gastrointestinal cancers, in particular pancreatic ductal adenocarcinoma (PDAC), is unfavorable when recognized at an advanced stage. The search for suitable biomarkers for the diagnosis, management and follow-up of gastrointestinal cancers is a relevant challenge of cancer research [1,2,3,4]. For several years, carbohydrate antigen 19.9 (CA19.9) has been widely used to address these aims in both research and clinical practice. It remains the serum pancreatic cancer marker against which new markers for this malignancy should be judged [5] and an unsubstituted tool necessary for any therapeutic choice for patients with advanced pancreatic cancer [6,7,8]. Many other molecules are indeed under investigation as alternatives or complements of CA19.9 [9,10,11]. 

**Figure 1:**
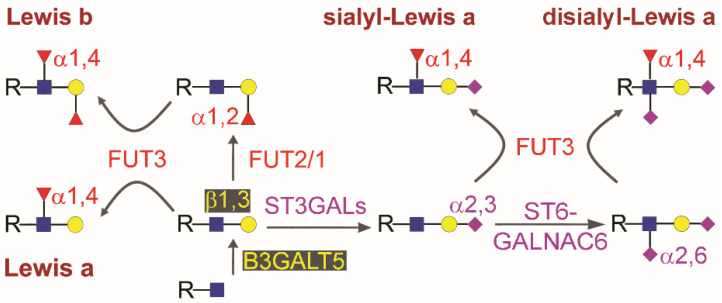
Structure of type 1 chain Lewis antigens and biosynthesis in gastrointestinal tissues. Monosaccharides are depicted according to the current representation: Blue square, GlcNAc, N-acetylglucosamine; yellow circle, Gal, galactose; Red triangle, Fuc, fucose; Pink diamond, sialic acid, Sia. Anomers, linkage positions, and enzymes involved in the reactions are indicated. Glycosyltransferases are named according to the HUGO recommendations. Note that B3GALT5 activity is necessary giving rise to the Galβ1,3GlcNAc-R sequence that is the common precursor of all the antigens, as is FUT3 activity, since the presence of a Fuc α1,4 linked to GlcNAc is mandatory for antibody recognition.

Very recently, the interest in CA19.9 has increased due to two independent observations. CA19.9 values are considered the best available at present for monitoring neoadjuvant therapy in PDAC, serving as a guide for clinical decision-making regarding the duration of neoadjuvant therapy and time to surgery [12]. Moreover, it was found that sialyl-Lewis a tetrasaccharide (sLea, Siaα2,3Galβ1,3[Fucα1,4]GlcNAc), the carbohydrate epitope of CA19.9 (see Figure 1 for the structure, biosynthesis, and involved enzymes), is a suitable target for developing therapeutic antibodies with anticancer activity [13,14,15]. This appears in line with the other recent finding that expression of sLea induces pancreatitis in a mouse model obtained by transgene expression of human fucosyltransferase FUT3 (a pseudogene in rodents) and galactosyltransferase B3GALT5, whose endogenous expression in mice was not sufficient to drive relevant CA19.9 synthesis. sLea-induced pancreatitis was found in turn able to accelerate cancer development in Kras-mutant mice [16]. 

CA19.9 is widely used as a marker in almost all other gastrointestinal cancers, including colon, gastric, and biliary tract cancers [17,18,19,20,21,22]. In spite of its many applications, the rationale underlying its use has never been addressed in detail and remains ambiguous. Is the circulating antigen produced by the cancer cells and not by their normal counterparts? Is the ability to produce CA19.9 common to cancer cells originating from different types of gastrointestinal tissues? Why is CA19.9 a tumor marker while the other type 1 chain Lewis antigens (see Figure 1 for the structures) are not? The answers to such basic biological questions should be obvious for a tumor marker, but they have never been clearly established for CA19.9. 

It has long been known that CA19.9 is present in various normal secretions, including seminal fluid [23], bile [24], and pancreatic juice [25]. In the pancreas, CA19.9 has appeared as a physiological secretory product that undergoes serum elevation in some cancer patients. It is generally believed that serum elevation of CA19.9 results from increased production by cancer cells. At least in colon cancer, this view is in sharp contrast to the finding that the enzymatic machinery is impaired in the synthesis of this antigen because of the down-regulation of the key enzyme B3GALT5 [26,27,28]. Thirty years ago, Kalthoff et al. [25] suggested that the high serum CA19.9 values found in pancreatic cancer depend on a mechanism involving reabsorption in the bloodstream due to the duct obstruction and inversion of polarity observed in ductal cells during malignant transformation [29]. Unfortunately, this hypothesis was not further investigated but rather was forgotten. 

CA19.9 represents a heterogeneous group of molecules whose epitope is sLea, recognized by the antibody NS-1116-19-9 [30] and originally discovered in a ganglioside [31]. The first study that described the antigen circulating in a colon cancer patient reported CA19.9 as a mucin [32], and recent proteomic data have shown that the molecules carrying the sLea epitope in patients affected by pancreatic diseases are various types of glycoproteins, including mucins, without relevant differences between malignant and nonmalignant pathological cases [33]. This is a crucial observation since it rules out a differential availability of the carrier molecules in pancreatic cancer. The nature of the molecules that carry the antigen secreted by other gastrointestinal organs has not yet been defined, and it may be even more heterogeneous, as suggested by a study performed in the bile [34].

In this perspective review we consider the recent progress in the biology of CA19.9 and related type 1 chain Lewis antigens, to better understand their origin in the blood of cancer patients. In particular, we tried to make the differences between the main gastrointestinal tissues clear since the data obtained in colon cancer do not apply to PDAC, and analogous information is currently unavailable for gastric and bile duct cancer. Moreover, we focus on the different clinical applications suggested by such data, which highlight the limits of such molecules as a tool for early diagnosis while stressing their potential role in the management and follow-up of some patients. Our purpose is to bring the molecular basis of CA19.9/Lewis antigen expression to the attention of pathologists, surgeons, and oncologists, which may help to address future directions in their use in translational research and clinical practice.

## 2. Biosynthesis of Type 1 Chain Lewis Antigens in Gastrointestinal Cancers

The sLea epitope is one of the so-called type 1 carbohydrate-chain Lewis antigens (Figure 1), which includes the Lewis a trisaccharide (Lea, Galβ1,3[Fucα1,4]GlcNAc), Lewis b tetrasaccharide (Leb, Fucα1,2Galβ1,3[Fucα1,4]GlcNAc), and disialyl-Lea pentasaccharide (Siaα2,3Galβ1,3[Fucα1,4][Siaα2,6]GlcNAc), in which the galactose residue is linked to N-acetylglucosamine through a β1,3 linkage. Due to the ability to act as selectin ligands, both sLea and the type 2 chain isomer sialyl-Lewis x (Siaα2,3Galβ1,4[Fucα1,3]GlcNAc, in which the galactose residue is linked to N-acetylglucosamine through a β1,4 linkage), have been extensively investigated in recent years (reviewed in [35,36]). Because of their capacity to enhance the malignant potential of cancer cells, as proven in model systems [36,37]), it is important to assess the actual expression of such structures by native cancers [38,39] compared to the normal counterpart. The ability to synthesize the various epitopes depends on the expression of specific glycosyltransferase enzymatic activities. In the case of the type 1 chain, two are mandatory: a β1,3 galactosyltransferase and an α1,4 fucosyltransferase. Although multiple gene products sharing such enzymatic activities are coded by the human genome, in vitro and in vivo data have suggested that between B3GALTs and FUTs, B3GALT5 and FUT3 are by far the two most relevant in gastrointestinal tissues [16,26], respectively. The expression levels of the other three glycosyltransferase activities, competing with each other, namely α2,3sialyltransferase, α1,2fucosyltransferase, and α2,6sialyltransferase, determine the amount of each antigen actually produced by a cell [40,41]. In this regard, *ST3GAL3*, *FUT2*, and *ST6GALNAC6* were proposed as the candidate genes, respectively. In particular, the expression of 

ST3GAL3 was considered to be relevant [42,43], since sialylation is crucial with respect to the cancer-associated synthesis of sLea. However, the exclusive role of ST3GAL3 has been definitely ruled out very recently in a study of two young children harboring a recessive non-sense mutation of the gene. They carry a totally inactive ST3GAL3 variant but do express circulating CA19.9 at high levels, according to their age [44]. This indicates that multiple ST3GALs independently contribute to synthesize the epitope. While the tissue and individual levels of each transcript are very heterogeneous, significant cancer-associated differences are not reported, except for ST6GALNAC6 [45] and, paradoxically, B3GALT5 [26,27,28], which is dramatically down-regulated in colon cancer. Notably, the glycosyltransferase pattern expressed in native tissues is available only for the colon [24,40,41,46,47] and pancreas [47,48,49], but not for other gastrointestinal organs, such as the stomach and the bile ducts (Table 1). Moreover, the available data suggest that the glycosyltransferase regulation is tissue-specific and affected by mechanisms that operate in only native tissues [28,50,51], making the studies performed in cell lines poorly representative.

## 3. Cancer Cells in Colon Cancer and Pancreatic Ductal Adenocarcinoma Do Not Overproduce CA19.9 

Previous studies indicated that pancreas and colon cancers expressed the antigen [52,53,54], while the other Lewis antigens were detected in normal colon mucosa [66], assumed as being a normal component of healthy tissues (Table 1). Positive reactions were frequently detected in both the cytoplasm of tumor cells and cancer stroma. Disialyl-Lewis a (the structure and biosynthesis are shown in Figure 1), originally isolated from a colon cancer metastasis [67], was then found expressed in normal colon mucosa and down-regulated in colon cancer, due to the down-regulation of cognate α2,6sialyltransferase ST6GALNAC6, leading to the conclusion that CA19.9 was specifically expressed in colon cancer due to the lack of the second sialylation step [45]. Interestingly, disialyl-Lea was found to act as a siglec ligand in normal colon mucosa [68]. The relevant observation concerning ST6GALNAC6 and disialyl-Lea downregulation in colon cancer was turned into the idea that CA19.9 was an abundant and specific product of all gastrointestinal cancer cells without any direct and confirmed evidence.

This commonly held view was serendipitously contradicted by an experimental study aimed at elucidating the malignant properties of colon cancer cells expressing sLea [65]. Immunohistochemical analysis of nude mice xenografts with the anti CA19.9 antibody NS-1116-19-9 revealed reactivity of mice tissues under the same conditions used for the detection in colon cancer [54]. This was in sharp contrast to the known absence of any of α 1,4fucosyltransferase activity or cognate type 1 chain Lewis antigen in mice [16,69], and suggested that immunohistochemical data alone could be unreliable. More recent data [47] obtained by dot-blotting and immunofluorescence indicated that, consistent with the down-regulation of B3GALT5, the CA19.9 antigen was almost undetectable in colon cancer. Conversely, the CA19.9 antigen was, to some extent, detectable in the normal mucosa where Lea was strongly expressed at the luminal surface of the cells. In the pancreas, the expression pattern of Lewis antigens CA19.9, Lea, and Leb was maintained in cancer, consistent with the similar expression of glycosyltransferase mRNAs detected in cancer and in the normal counterpart. In particular, CA19.9 expression was restricted to the luminal surface of the ducts or ductal-like structures, while it was absent in the bulk of cancer cells and displayed marked individual differences in expression. No cancer-associated deregulation of specific glycosyltransferases was found in the pancreas, nor altered synthesis or secretion of the carrier molecules [33], despite the presence of antigen accumulation, as determined by dot-blotting of tissue lysates [47].

Further relevant data came from the study of hematological malignancies arising in the pancreas [70]. Surprisingly, an elevation of serum CA19.9 values is common in these cancers although they usually lack expression of type 1 chain Lewis antigens. On average, the levels of CA19.9 expression in these hematological malignancies are lower but partially overlap those of adenocarcinomas. Moreover, in a nonmalignant disease such as chronic pancreatitis, endoscopic retrograde choledocho-pancreatography revealed that a stronger elevation in serum CA19.9 correlated with the obstruction of the main pancreatic duct [56]. Altogether, these results support the hypothesis that elevation of circulating antigens in the serum of pancreatic cancer patients depends on the obstruction of the ducts and the loss of polarity of the ductal cells, as was proposed long ago [25].

## 4. A Proposed Model of Circulating CA19.9 and Type 1 Chain Lewis Antigen in Pancreatic Ductal Adenocarcinoma (PDAC)

Taking into account the above data and hypothesis, no specific association exists in the pancreas between cancer and CA19.9 with respect to Lea or Leb because each can be reabsorbed into the blood by individual patients. Only if the normal pancreatic ducts express and secrete high levels of CA19.9, upon malignant transformation does the bloodstream reabsorb large amounts of CA19.9. Rather, if the normal ducts mainly express and secrete Leb, or Lea, but low levels of CA19.9, upon malignant transformation the bloodstream cannot reabsorb relevant amounts of CA19.9, but probably does reabsorb Leb or Lea instead. According to this model, a proportion of pancreatic cancer patients that are negative for circulating CA19.9 are expected to be positive for Leb or Lea, while those where obstruction and/or loss of polarity do not occur are expected to remain negative for all such antigens. 

Detection of CA19.9 in the serum or other fluids or tissue homogenates is easily performed by a sandwich Enzyme-linked immunosorbent assay (ELISA) [44] using immobilized and labeled anti-CA19.9 antibody as the capture and detection antibody, respectively. We have set a similar procedure for Lea and Leb using the corresponding antibodies purified from available hybridomas (anti-Lea 151–6-A7–9, ATCC HB 8324, and anti-Leb 130–3-F7–5, ATCC HB 8326) and screened up to 20 sera collected from healthy volunteers, obtaining a range of values spanning from unmeasurable to an arbitrary maximum level, which was assumed as a reference putative normal range for each antigen. More recently, we collected the serum from 10 PDAC patients judged suitable for surgical resection and performed ELISAs under the conditions set for the healthy group. Five patients displayed values inside or close to the control range, while 5 patients displayed higher values: 3 patients for Lea only, 1 patient for both CA19 and Leb, and 1 patient for all three antigens. In 1 of the 3 patients that expressed higher values for Lea only, the value was 120 times higher than the highest values detected in the control group (unpublished data from the authors). Although very preliminary, these data indicate that detection of circulating Lea and Leb is simple and available as that of CA19.9. 

In the blood of pancreatic cancer patients, the concentrations of CA19.9, Lea, and Leb antigens appear to depend on the following two main factors: (1) the reabsorption of the antigens by the circulating blood, which, in turn, is probably a side effect of the duct obstruction and/or loss of polarity; and (2) the production of each antigen by normal pancreatic ducts. Consequently, the following two crucial aspects need to be investigated in more detail: the obstruction and loss of polarity, which has been poorly investigated so far in gastrointestinal tumors [71] but has already been studied in other organs [72,73,74], and the individual expression pattern of antigens, which is the result of the expression of genetically and epigenetically regulated glycosyltransferases, namely FUT1-3 and B3GALT5. 

Null alleles of *FUT3* and *FUT2* are present in the human population, and their genomic dosage affects the amounts of individual antigens expressed, including circulating CA19.9 [75,76,77,78]. FUT3^−/−^ subjects are known to lack any type 1 chain Lewis antigen, while FUT3^+/+^/FUT2^−/−^ individuals are candidates for higher expression of CA19.9 than FUT3^−/+^/FUT2^+/+^ individuals. Expression of B3GALT5 is controlled by two main epigenetically regulated promoters, whose combined or alternative usage gives rise to strong individual differences of expression [28,47,50], and the level of B3GALT5 affects the synthesis of the antigens similarly to FUT3 [16]. Profiling the glycosylation potential of each patient is thus useful for a critical use of such antigens as tumor markers. The choice of eligible antigen(s) and its/their cutoff value of expression should be defined for each patient on the basis of the personal glycosylation machinery. Consistent with this view, in pancreatic cancer patients lacking elevation of CA19.9 other Lewis antigens may be used as an alternative for follow-up and surveillance, including sialyl-Lewis x [79,80]. 

## 5. CA19.9, Lea, and Leb in Colon Cancer and Other Gastrointestinal Pathologies 

The situation is different in colon cancer (Table 1), where the dramatic down-regulation of B3GALT5 results in undetectable type 1 chain antigens, particularly CA19.9. In this case, increased expression of the type 2 chain antigen sialyl-Lewis x has been proposed [81] as the consequence of a colon-specific down-regulation of β1,4N-acetyl-galactosaminyltransferase B4GALNT2, which synthesizes the alternative carbohydrate antigen Sda [82]. Since elevation of circulating CA19.9 in patients occurs infrequently and only in the late stage of the disease [17,22], we hypothesize that the circulating antigen is not derived from the cancer but from other sources [62]. The biliary system is an outstanding candidate since bile contains huge amounts of antigen and patients with high serum CA19.9 are very frequently jaundiced. Coherently, serum CA19.9 elevation is frequently reported in jaundiced patients suffering benign diseases [63,64]. Bile duct obstruction commonly occurs in colon and other gastrointestinal cancers, and a mechanism of polarity reversal has been detected in bile duct cells, where it appears to be responsible for a kind of non-secretory exocytosis involving vesicles that carry CA19.9 [26]. In this case, the serum levels of Lewis antigens could be independent from those of bilirubin. In this context, it is also possible to speculate that elevation of serum CA19.9 may be an early functional sign of colon cancer metastasis affecting the liver. It is worth noting that several non-malignant diseases of the liver and biliary system are accompanied by increased values of circulating CA19.9. They include hepatitis, gallstones, cholelithiasis, or even autoimmune diseases [83,84]. Increased serum CA19.9 values are also commonly found in the inflammatory diseases of the pancreas [85,86] and the kidney [87,88]. These evidences furtherly suggest that CA19.9 expression and secretion is not a signature of cancer cells.

The clinical use of CA19.9 as a tumor marker is also widely reported in diseases of the stomach [58,59,60] and biliary ducts [55,61], sometimes due to the lack of alternatives. Molecular data concerning these two organs are lacking, and those available from colon and pancreas suggest that the molecular bases of CA19.9 expression are tissue specific and cannot be predicted without dedicated experimental work. Moreover, activation of other *B3GALT* genes, as reported in prostatic cancer [89], cannot be excluded. Very recently, an unusual carrier of sLea with potential therapeutic relevance has been detected in gastric cancer cell lines [90], which deserves confirmation in native tissues. We suggest that the future use of Lewis antigens as tumor markers should be based on knowledge of their expression pattern in native cancers and related normal tissues, as well as in nonmalignant diseases of the same organs. 

## 6. Future Applications and Limit of Type 1 Chain Lewis Antigens as Personalized Tumor Markers in Gastrointestinal Cancers

Medical associations have provided rather restrictive recommendations about CA19.9, suggesting that it should be used as a tumor marker only for the management of pancreatic cancer, but not for its early diagnosis or for other gastrointestinal cancers [5,91]. 

According to our working hypothesis, a solid biological background exists for such recommendations and suggests that the other related Lewis antigens, Lea and Leb, should be considered together with CA19.9 when its use is recommended. There is no reason to measure Lea and Leb when CA19.9 determination is not appropriate. In fact, Lea, Leb, and CA19.9 share a common origin, which is the normal epithelium of the gastrointestinal tract, at least of pancreatic, bile ducts, and colon mucosa. At present, the lack of validated data does not permit drawing conclusions about the stomach. Their amounts in the bloodstream appear to follow different mechanisms. Colon cancer cells down regulate the biosynthesis of all such antigens due to the silencing of B3GALT5. In colon cancer, they become detectable in patient serum only at late stages, presumably as a consequence of tumor spreading and metastasis. In PDAC, serum elevation probably occurs when duct obstruction and a loss of polarity affect a relevant tumor mass. In both cases, there is no rationale for their use in early diagnosis. Their serum levels are expected to remain at the baseline in the early stage of the disease, as happens for CA19.9, when the tumor mass with obstructed ducts and reverse in polarity is small. Moreover, Lea and Leb could not increase the specificity of CA19.9, which remains unfortunately low. Their serum levels are expected to be elevated even in non-malignant obstructive diseases, i.e., pancreatitis, as happens with CA19.9, without improving the differential diagnosis. Conversely, they are able to help define the resectability of the tumor, monitoring neoadjuvant therapy, evaluating the response to therapy, and predicting recurrences, as much as CA19.9 [8,92]. More importantly, they can do that in patients lacking CA19.9 elevation, which is even more difficult to manage [57].

Epidemiological surveys have reported that the number of serum CA19.9 determinations routinely performed in some Western countries is enormous, largely exceeding the number expected on the basis of the prevalence of the diseases for which the CA19.9 assay is recommended by the scientific literature [93,94]. We believe that this is due to the use of the antigen for diagnosis when gastrointestinal cancer is suspected. Such an approach lacks a solid biologic background and should be avoided or cautiously pursued in the future.

Conversely, we invite pathologists to evaluate the actual expression of CA19.9, Lea, Leb, and sialyl-Lewis x in tissue sections of surgical specimens derived from both cancer and surrounding health resection margins. We also invite surgeons and oncologists to measure circulating Lea and Leb in addition to CA19.9, or to keep serum aliquots available for the assay in patients diagnosed with gastrointestinal malignancies before surgery, during neoadjuvant therapy when applicable, and during post-surgery follow-up and treatments if performed. 

We speculate that the origin of these circulating antigens depends on the type of gastrointestinal cancer and is strictly related to the tissue pattern of carbohydrate antigens and to the tissue-specific regulation of glycosyltransferases. Assessment of the glycosylation potential of each tissue at the molecular level could also be useful, including determination of FUT3 and FUT2 allelic status relative to common mutations. This could be performed on surgical resections and/or bioptic specimens. By comparing the glycosylation pattern of the healthy resection margins with that of the cancer in the context of serum and clinical data, it should be possible to rationalize the use of the antigens for both research and clinical purposes in a personalized perspective.

## 7. Conclusions

**Figure 2:**
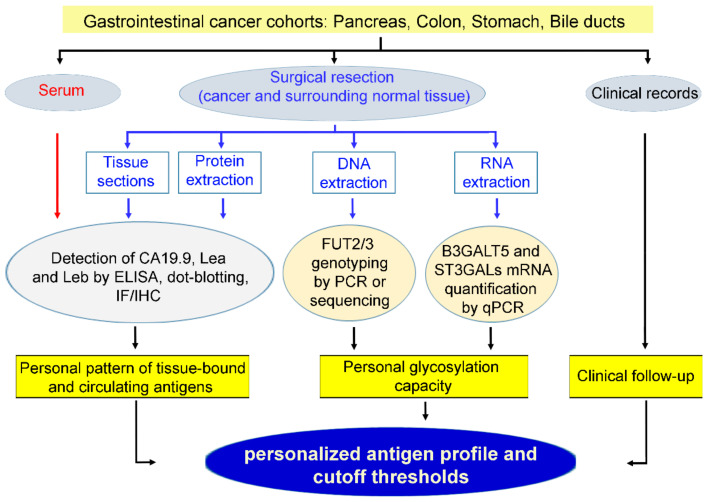
Flowchart of the approach proposed for using Lewis type 1 chain antigens as personalized tumor markers. IF: immunofluorescence; IHC: immunohistochemistry. Our working hypothesis is that the epitope of the CA19.9 antigen is not overproduced in cancer cells nor distinct from the related carbohydrate antigens Lea and Leb in a cancer-associated manner. Consequently, they should be considered together, taking into account their frequent expression, which is sometimes abundant in normal tissues. At the molecular level, the concurrent amounts of two transcripts, FUT3 and B3GALT5, appear highly predictive of the expression levels of all three antigens, while the levels of FUT2/1 and ST3GALs affect the relative quantity of each individual antigen. At present, complete and homogeneous data are not available in human samples, and those found in cell lines are not predictive. Transcript and antigen expression in normal colon mucosa and colon cancer is well documented (Table 1), while parallel data on the antigen expression in patient serum are not available.

Recent advances in many fields clearly indicate that the future of medicine is the personalization of diagnostic approaches and therapies. For example, the efficacy of highly specific biological drugs, including monoclonal antibodies and kinase inhibitors, is totally dependent on the presence of their molecular targets, which are expressed only by subsets of patients who consequently should be distinguished from those affected by an apparently identical disease lacking the target molecule. In light of the biological basis of CA19.9 expression, we propose to extend the idea of personalized medicine to the use of Lewis type carbohydrate antigens as tumor markers in gastrointestinal cancers (Figure 2). 

In the normal pancreas ducts, B3GALT5 is not overabundant with respect to FUTs and ST3GALs nor downregulated in PDAC, and the transcripts show individual heterogeneity. Published results (Table 1) and our preliminary data here reported (see Section 4) suggest a parallel expression of Lewis antigens in both normal and cancer ducts, and a frequent cancer-associated elevation of one or more of such antigens in the patient serum. This appears to be the most promising field of application of CA19.9, Lea, and Leb, as complementary markers for managing PDAC at various stages. Conversely, the biological background depicted above does not support a rationale for using CA19.9, Lea, and Leb for the early or differential diagnosis of PDAC or colon cancers.

The extension of similar studies to other gastrointestinal cancers, namely gastric and bile duct cancers, is still necessary to explore the potential utility of such antigens in these patients.

We have suggested a novel conceptual frame underlying serum CA19.9 and type 1 chain antigen elevation in gastrointestinal cancers, where the individual glycosyltransferase pattern and regulation play a pivotal role in a tissue-specific manner. We believe that 30 years after their first use the story of CA19.9 and its related antigens still promises fruitful clinical applications.

## Figures and Tables

**Table 1:** Features of type 1 chain Lewis antigens in gastrointestinal cancers.

Site	Topic	Findings	Reference
Colon	Glycosylation profile	High B3GALT5 ^1^ in normal mucosa, downregulated in cancer, low ST3GALs and high FUT3 in both ST6GALNAC6 downregulated in cancer	[26,27,28,47] [40,46,47] [45]
	Tissue antigens by immunofluorescence/dot-blot	High Lea ^2^ and very low CA19.9 and Leb ^3^ in normal mucosa (luminal staining), none detected in cancer	[47]
	Tissue antigens by immunohistochemistry	CA19.9 more elevated in cancer (frequent stromal and cytoplasmic staining) but present in normal mucosa and benign polyps, Lea and Leb abundant in normal mucosa disialyl-Lea more elevated in normal mucosa but present in cancer	[45,52,53,54][45,53]
	Serum	CA19.9 elevated in cancer at low percentage and late stages only; no data available for Lea and Leb	[17,22]
Pancreas	Glycosylation profile	B4GALT5, FUT3, FUT1/2 and ST3GALs well detectable in both normal tissue and PDAC ^4^	[47,48,49]
	Tissue antigens by immunofluorescence/dot-blot	CA19.9, Lea, and Leb detected in both normal and cancerous ducts (luminal staining), no one in normal acinar cells or poorly differentiated cancer cells	[47]
	Tissue antigens by immunohistochemistry	CA19.9 detected in cancer (frequent cytoplasmic staining) better than in normal ducts (luminal staining)	[52,53]
	Serum	CA19.9 elevation in PDAC Lea and Leb elevation in PDAC (preliminary) CA19.9 elevation in non-malignant diseases	[4,7,12,55] [this article] [56,57]
Stomach	Glycosylation profile	No data available	
	Tissue antigens by immunofluorescence/dot-blot	No data available	
	Tissue antigens by immunohistochemistry	CA19.9 detected in both cancer and normal mucosa, frequent cytoplasmic and stromal staining; no data about Lea and Leb	[52,53,58]
	Serum	CA19.9 elevation in cancer, no data about Lea and Leb	[58,59,60]
Bile ducts	Glycosylation profile	No data available	
	Tissue antigens by immunofluorescence/dot-blot	No data available	
	Tissue antigens by immunohistochemistry	CA19.9 detected in both cancer and normal ducts No data about Lea and Leb	[52,61]
	Serum	CA19.9 elevation in both cancer and non-malignant diseases No data about Lea and Leb	[21,55,62,63,64]
Mouse tissues	Tissue antigens by immunofluorescence	CA19.9 undetectable in liver, colon, and HCT-15 ^5^ xenograft; HCT-15-T5 ^6^ xenograft very bright	[65]
	Tissue antigens by immunohistochemistry	CA19.9 detected in liver, colon, adipose tissue, small intestine and both xenografts (poorly, including stromal staining)	

^1^ Glycosyltransferases are named according to the HUGO recommendations. *^2^* Lea, Lewis a antigen; ^3^ Leb, Lewis b antigen. ^4^ PDAC, pancreatic ductal adenocarcinoma. ^5^ HCT-15 xenograft derives from the injection of the parental HCT-15 cell line that lacks CA19.9; ^6^ HCT-15-T5 xenograft derives from a recombinant clone that expresses CA19.9.
